# Maternal Caffeine Consumption during Gestation and Lactation Abolishes Cortical Oxidative Stress and Restores Na^+^/K^+^-ATPase Activity in Neonates Exposed to Hyperthermia-Induced Seizures

**DOI:** 10.3390/biomedicines11123292

**Published:** 2023-12-12

**Authors:** María Crespo, David Agustín León-Navarro, Mairena Martín

**Affiliations:** 1 Department of Inorganic, Organic Chemistry and Biochemistry, Faculty of Chemical and Technological Sciences, Regional Centre of Biomedical Research (CRIB), Universidad de Castilla-La Mancha, 13071 Ciudad Real, Spain; 2 Department of Inorganic, Organic Chemistry and Biochemistry, Faculty of Chemical and Technological Sciences, School of Medicine of Ciudad Real, Regional Centre of Biomedical Research (CRIB), Universidad de Castilla-La Mancha, 13071 Ciudad Real, Spain; mairena.martin@uclm.es

**Keywords:** Na^+^/K^+^ ATPase, Mg^2+^ ATPase, oxidative stress, neonates, cortex, febrile seizure

## Abstract

Caffeine is a psychoactive substance that is widely consumed by individuals of various demographics, including pregnant women. It can readily cross the blood–brain and placental barriers, easily reaching the fetal brain. In addition, caffeine has also shown antioxidant properties, as its consumption reduces oxidative stress in various pathologies, including epilepsy. Febrile seizures (FS) are among the most common convulsive disorders in infants and young children. Here, we used an animal model of FS to learn whether maternal caffeine (1 g/L) intake consumption during gestation and lactation could exert beneficial effects on the rat cortex. Neonatal development was analyzed by measuring pinna opening, eye opening, righting reflex on the surface, and geotaxis reflex. Five and twenty days after HIS, the rats were euthanized, and plasma membranes and cytosolic fractions were isolated from their cortex brain. The enzymatic activities of glutathione reductase, glutathione S-transferase, Na^+^/K^+^-ATPase, and Mg^2+^-ATPase, as well as the levels of thiobarbituric acid reacting substances, were quantified. Results showed that maternal caffeine intake eliminates oxidative stress and normalizes Na^+^/K^+^-ATPase activity disrupted by HIS and also affects some parameters relating to the neurodevelopment of neonates. As FS in infants has been related to epilepsy in adults, the antioxidant properties of caffeine could prevent potential damage from hyperthermia.

## 1. Introduction

Caffeine is a psychoactive substance widely consumed by different people, including pregnant women [1]. The metabolism of this substance is modified during pregnancy, resulting in an increased half-life [2]. As a result, caffeine can readily cross the blood–brain barrier and the placental barrier, easily reaching the fetal brain [3,4]. Additionally, it has been demonstrated that maternal caffeine consumption during lactation can transfer caffeine to the neonatal brain through breast milk [5].

Caffeine also has antioxidant properties [6], and its consumption has been demonstrated to reduce oxidative stress in various pathologies, including epilepsy [7,8,9]. Febrile seizures (FS) are the most common neurological disorder in childhood [10,11]. FS are classified into simple (t < 15 min) and prolonged febrile seizures (t > 15 min) based on their duration. Generally, simple FS have a good prognosis, while prolonged FS present more uncertainties. Although retrospective epidemiological studies have suggested an association between this type of seizure and the later onset of epilepsy in adulthood, prospective studies have not confirmed such a relationship [12,13]. To address these discrepancies and gain insights into the molecular effects caused by FS, various animal models have been developed. One of the most extensively utilized models is the hairdryer model, which was developed by Baram and coworkers [14]. In this model, hyperthermia-induced seizures (HIS) are elicited in neonatal rats using an adjustable stream of heated air. Different results obtained in our research group have led us to consider that febrile seizures (FS) may induce oxidative stress in the cerebral cortex and play a significant role in the epileptogenic process. For instance, we previously demonstrated that FS induced oxidative stress in the cerebellum in the short term [15]. Additionally, we found that FS altered the functionality of Na^+^/K^+^-ATPase, a critical pump involved in the regulation of neuronal excitability [16] in the cortex brain. In addition, it has been shown that oxidative stress can alter the activity of this pump [17]

Therefore, the objectives set in this study were as follows: (a) to determine whether febrile seizures (FS) induced oxidative stress in the neonatal cortex; (b) to investigate whether maternal caffeine consumption could attenuate this oxidative stress; and (c) to examine whether maternal caffeine consumption could reverse the modulation of Na^+^/K^+^-ATPase and Mg^2+^-ATPase activities observed in neonates exposed to FS.

## 2. Materials and Methods

### 2.1. Materials

Adenosine triphosphate (ATP), 1-chloro-2,4-dinitrobenzene (CDNB), L-Glutathione oxidized, NADPH, L-Glutathione reduced, thiobarbituric acid, and malachite green were from Sigma-Aldrich (Madrid, Spain). Ammonium molybdate was from Merck (Madrid, Spain). All other reagents were of analytical grade and were obtained from commercial sources.

### 2.2. Animals

The care and use of animals were carried out in accordance with the European Directive 2010/63/EU and Spanish laws (RD 53/2013 and Ley 32/2007) governing the use of laboratory animals. All experiments were approved by the Animal Experimental Committee of the University of Castilla-La Mancha (EXP 26/2014/13) (approved on 3/12/2014). Every effort was made to minimize animal suffering and reduce the number of animals used. The animals were kept in a temperature-controlled room (25 °C) with a 12-h light/12-h dark cycle (lights on at 07:00 h) and constant humidity (40–50%) and were provided with free access to food and drinking water.

For this study, two groups of pregnant Wistar rats were utilized. The first group received caffeine (1 g/L) in their drinking water from gestational day 2 throughout gestation and lactation. This dosage was chosen based on previous research indicating that caffeine could readily pass through the placental and brain–blood barriers and could also be transmitted to the neonatal brain via maternal milk [5,17]. The second group of pregnant rats received regular tap water.

On postnatal day 13, half of each litter was subjected to hyperthermia-induced seizures, while the other half served as controls. Five (PD18) and twenty days after the hyperthermic insult (PD33), the rats were euthanized, and their cortical brains were removed to investigate the effects of maternal caffeine consumption and hyperthermia-induced seizures on oxidative stress parameters, Na^+^/K^+^-ATPase, and Mg^2+^-ATPase activities. The pups were housed with their mothers until weaning at postnatal day 21. Therefore, four groups of animals were used in this work:

(a) Water control rats: these animals were not exposed to HIS, and their mothers drank water during gestation and lactation;
(b) Water HIS rats: these animals were exposed to hyperthermic seizures, and their mothers drank water during gestation and lactation;
(c) Caffeine control rats: these animals were not exposed to HIS, and their mothers drank caffeine during gestation and lactation;
(d) Caffeine HIS rats: these animals were exposed to HIS, and their mothers drank caffeine during gestation and lactation.

### 2.3. Hyperthermia-Induced Seizures

On postnatal day 13 (PD 13), neonatal rats were exposed to hyperthermia-induced seizures (HIS). This developmental stage aligns with a human age equivalent to being from several months to 3 years old, a period highly susceptible to febrile seizures. Hyperthermia was induced using a hair dryer positioned 50 cm above a plastic chamber (17 cm × 12 cm × 12 cm) with a warmed air stream (45–50 °C), following a previously established protocol [15]. Rectal temperature was monitored at 2-minute intervals.

Seizure occurrence was assessed by two observers monitoring the rats’ behavior. The observed behavioral seizures, previously correlated with electroencephalography (EEG) discharge [14], included the cessation of heat-induced hyperkinesia, followed by body flexion. All pups exhibited these behaviors, along with rearing and falling, indicative of hind-limb clonus seizures (stage 5 on the Racine scale criteria), approximately 10 min after hyperthermia induction when the core temperature reached around 42 °C.

After 20 min of behavioral seizures, the pups were moved to a cool surface until their rectal temperature returned to baseline. Subsequently, they were returned to their home cages with their mothers. The heating duration lasted for 27 ± 3 min. The control group, separated from their mothers for the same duration, was placed in a chamber at room temperature.

### 2.4. Preparation of Plasma Membranes and Cytosolic Fractions from Rat Cortex

Rat cortical brains were homogenized in an isolation buffer, which consisted of 50 mM Tris-HCl (pH 7.4) with 10 mM MgCl_2_ and protease inhibitors. The homogenization was performed using a Dounce homogenizer, with pestle A for 10 strokes and pestle B for an additional 10 strokes. After homogenization, the preparations were centrifuged for 5 min at 1000× *g* in a Beckman JA 21 centrifuge (Coulter España, Madrid, Spain). This step separated the supernatant from the cellular components. The obtained supernatant was further centrifuged for 10 min at 27,000× *g*. This step aimed to separate the plasma membrane fraction (pellet) from the cytosolic fraction (S2). The pellet containing the plasma membrane fraction was finally resuspended in the isolation buffer. The protein concentration in the isolated fractions was determined using the Lowry method, with bovine serum albumin (BSA) serving as the standard for calibration.

### 2.5. Neonatal Development

A neonatal development assessment was conducted to investigate potential alterations resulting from maternal caffeine consumption. Thirty-two neonates (10 controls and 22 exposed to maternal caffeine) were used. The following parameters were measured:

(a) Auricle opening: the postnatal day when the auricles were fully opened was recorded;
(b) Righting reflex on the surface: pups were placed face up on a smooth surface, and the time it took for them to assume a normal position was noted. The animals were allowed 15 s to turn over;
(c) Geotaxis reflex: pups were positioned on an inclined surface with their heads facing downward, and the time required for them to reorient and face upward was recorded. The animals were given 30 s to perform the task;
(d) Eye opening: the day when the pups first opened their eyes was recorded.

### 2.6. Lipid Peroxidation Levels Determinations in the Cortex of Rats Submitted to Hyperthermia-Induced Seizures

The effect of HIS on lipid peroxidation levels was analyzed by measuring the thiobarbituric acid reacting substances (TBARS) in the following homogenates:

(a) Five days after HIS: control water (*n* = 3); HIS water (*n* = 4); control caffeine (*n* = 5); and HIS caffeine (*n* = 3);
(b) Twenty days after HIS: control water (*n* = 5); HIS water (*n* = 4); control caffeine (*n* = 3); and HIS caffeine (*n* = 5).

We followed the protocol previously described [15] with minor modifications. In this regard, 20 μL of homogenates were mixed with 0.5 mL of 10% trichloroacetic acid and 0.5 mL of 0.67% thiobarbituric acid. The mixture was then boiled at 100 °C for 15 min. Subsequently, 0.5 mL of butanol was added to the solution. Following centrifugation (4000× *g*, 5 min), the TBARS were determined by measuring the absorbance at 535 nm in the supernatant. The results are expressed as nmol/g tissue.

### 2.7. Glutathione Reductase Activity

Glutathione reductase (GR) activity was measured in the following samples:

(a) Five days after HIS: control water (*n* = 5); HIS water (*n* = 5); control caffeine (*n* = 5); and HIS caffeine (*n* = 4);
(b) Twenty days after HIS: control water (*n* = 5); and HIS water (*n* = 5).

We followed the protocol described in the Glutathione Reductase Assay Kit from Sigma (catalog number GRSA). Briefly, GR activity was assessed in accordance with the protocol outlined in the Glutathione Reductase Assay Kit from Sigma (catalog number GRSA). The assay utilized 80 μL of crude cytosolic fraction in a final volume of 1 mL of 100 mM potassium phosphate buffer (pH 7.5), which included 1 mM EDTA, 1 mM oxidized glutathione, and 0.1 mM NADPH at 25 °C. Enzymatic activity was determined by measuring the reduction in NADPH absorbance at 340 nm and expressed as micromoles of oxidized NADPH per milligram of protein per minute. The calculations employed the molar extinction coefficient for NADPH, which was set at 6.22 mM^−1^ cm^−1^.

The assay was performed using 80 μL of crude cytosolic fraction in a final volume of 1 mL of 100 mM potassium phosphate buffer (pH 7.5) containing 1 mM EDTA, 1 mM oxidized glutathione, and 0.1 mM NADPH at 25 °C. The enzymatic activity was quantified by measuring the decrease in NADPH absorbance at 340 nm and calculated as μmol of oxidized NADPH per milligram of protein per minute, using the molar extinction coefficient for NADPH of 6.22 mM^−1^ cm^−1^.

### 2.8. Glutathione S-Transferase Activity

Glutathione S-Transferase (GST) activity was measured in the following samples:

(a) Five days after HIS: control water (*n* = 5); HIS water (*n* = 5); control caffeine (*n* = 5); and HIS caffeine (*n* = 4);
(b) Twenty days after HIS: control water (*n* = 4); and HIS water (*n* = 5).

We followed the protocol described in the Glutathione Reductase Assay Kit from Sigma (catalog number CS0410). In summary, the assay utilized 40 μL of cytosolic fraction in a final volume of 1 mL of Dulbecco’s phosphate-buffered saline, which included 1 mM CDNB (1-Chloro-2,4-dinitrobenzene) and 2 mM L-Glutathione Reduced maintained at 25 °C. The reactions were initiated by the addition of the cytosolic fraction, and absorbance was recorded over a 5-minute period at 340 nm. Enzymatic activity was determined using the molar extinction coefficient for the CDNB-GSH conjugate, established at 9.6 mM^−1^·cm^−1^. The activity was expressed as micromoles per milligram of protein per minute.

### 2.9. Na^+^/K^+^-ATPase and Mg^2+^-ATPase Activities Assay

Enzymatic activities were analyzed in the following samples:

Na^+^/K^+^-ATPase activity:

(a) Five days after HIS: control water (*n* = 5); HIS water (*n* = 5); control caffeine (*n* = 4); and HIS caffeine (*n* = 4);
(b) Twenty days after HIS: control water (*n* = 7); HIS water (*n* = 7); control caffeine (*n* = 5); and HIS caffeine (*n* = 4).

Mg^2+^-ATPase activity:

(a) Five days after HIS: control water (*n* = 4); HIS water (*n* = 4); control caffeine (*n* = 5); and HIS caffeine (*n* = 4);
(b) Twenty days after HIS: control water (*n* = 7); HIS water (*n* = 7); control caffeine (*n* = 5); and HIS caffeine (*n* = 3).

Enzyme activities were assessed using the methodology detailed previously [16]. The reaction mixture comprised 5.0 mM MgCl_2_, 80.0 mM NaCl, 20.0 mM KCl, and 40.0 mM Tris–HCl (pH 7.4), with a final volume of 200 μL. Initiation of the reaction occurred through the addition of 3 mM ATP. Mg^2+^-ATPase activity was quantified with the inclusion of 1.0 mM ouabain. Na^+^/K^+^-ATPase activity was determined as the disparity between the total activity and the Mg^2+^-ATPase activity. The enzyme’s specific activity was expressed as nanomoles of inorganic phosphate (Pi) released per minute per milligram of protein.

### 2.10. Statistical and Data Analysis

Statistical analyses were conducted employing an unpaired two-tailed Student’s *t*-test and a two-way ANOVA, followed by a Bonferroni post hoc comparison test using GraphPad Prism 5. The findings are presented as mean ± standard error of the mean, and statistical significance between mean values was determined at *p* < 0.05.

## 3. Results

### 3.1. Effect of Chronic Maternal Caffeine Intake on Neonatal Development

**Figure 1:**
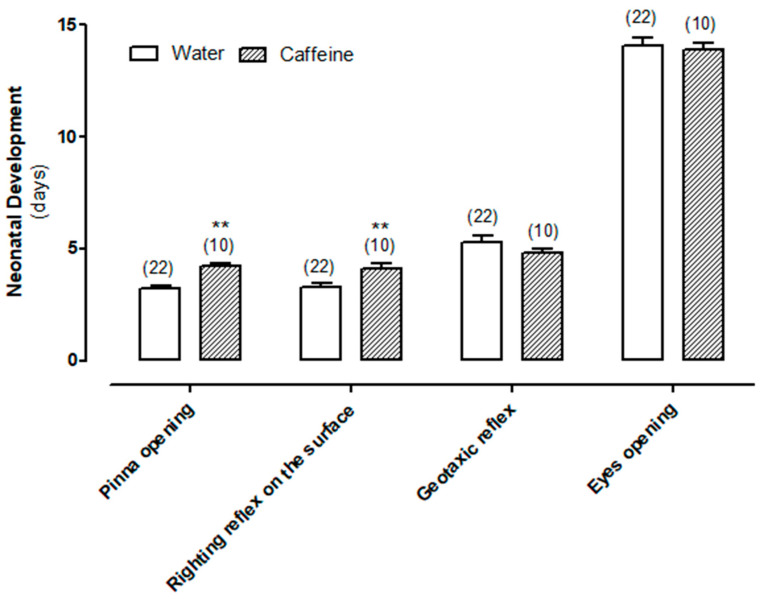
Effect of chronic maternal caffeine (1 g/L) consumption during gestation and lactation on neurodevelopmental parameters. Values are the mean ± S.E.M. of 10–22 animals (in parentheses) in each group. ** *p* < 0.01 significantly different from control group, according to Student’s *t*-test.

To assess the potential impact of chronic maternal caffeine intake on neonatal development, we compared the onset of various parameters between the group of neonates exposed to maternal caffeine and the control group whose mothers consumed water. As shown in Figure 1, pinna opening (3.2 ± 0.1 vs. 4.2 ± 0.1 days, *p* < 0.01) and righting reflex on the surface (3.3 ± 0.2 vs. 4.1 ± 0.2 days, *p* < 0.01) were significantly delayed in the caffeine-treated group. However, no significant differences were observed when the geotaxic reflex and eye opening were analyzed.

### 3.2. Effect of HIS and Maternal Caffeine Intake during Gestation and Lactation on TBARS Level in Cortex Brain

**Figure 2:**
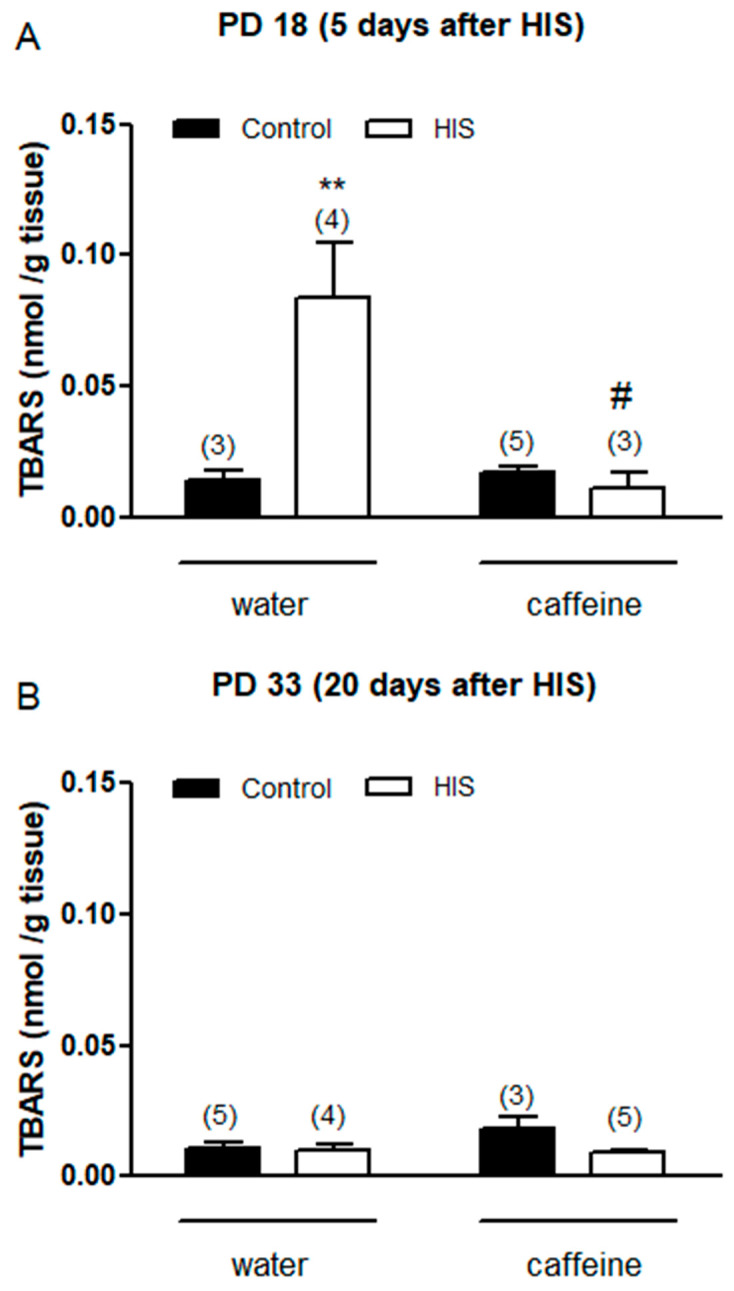
The impact of hyperthermia-induced seizures (HIS) and maternal caffeine consumption (1 g/L) during gestation and lactation on TBARS levels in the neonatal cortex (PD18 and PD33) was assessed at 5 (**A**) and 20 days (**B**) post hyperthermia-induced seizures. The bar graphs depict the influence of HIS on TBARS levels measured in the cortex of neonates born to mothers who consumed either water or caffeine during gestation and lactation. The data represent mean ± S.E.M. values of 3–5 neonates (in parentheses) from independent litters. ** *p* < 0.01 indicates a significant difference from the corresponding water control group, and # *p* < 0.05 indicates a significant difference from the water HIS group, as determined with two-way ANOVA and Bonferroni post hoc test. The control group comprises pups not exposed to hyperthermic stimulus but separated from the dams for the same duration and placed in a chamber at room temperature. HIS denotes pups subjected to hyperthermia-induced seizures as described in Methods.

Thiobarbituric acid reactive substances (TBARS) are considered a good index of lipid peroxidation and oxidative damage. For this reason, we wanted to evaluate the impact of HIS (one factor) and chronic maternal caffeine intake (second factor) on TBARS levels 5 days after hyperthermic seizures. A two-way ANOVA analysis revealed that there was a significant effect of HIS [F(1,11) = 7.1, *p* = 0.02], caffeine [F(1,11) = 8.5, *p* = 0.014], and HIS × caffeine interaction [F(1,11) = 10.01, *p* = 0.009]. The Bonferroni post-test showed that HIS significantly increased the TBARS level in control rats (*p* < 0.01), and this effect was significantly avoided with caffeine (*p* < 0.05) (Figure 2A).

Next, we wanted to analyze the impact of both factors on TBARS level 20 days after hyperthermic seizures. In this case, two-way analyses did not find any significant effect of HIS [F(1,13) = 4.4, *p* = 0.055], caffeine [F(1,13) = 1.6, *p* = 0.22, or HIS × caffeine interaction [F(1,13) = 2.84, *p* = 0.11] (Figure 2B).

### 3.3. Effect of HIS and Maternal Caffeine Intake during Gestation and Lactation on Glutathione Reductase Activity in Cortex Brain

Once we learned the effects of both HIS and maternal caffeine intake on TBARS levels in the cortex brain, we decided to analyze the impact of both factors on glutathione reductase (GR) and glutathione-S-transferase (GST) activities since both enzymes were key in cellular redox homeostasis. Thus, glutathione reductase is crucial in sustaining the levels of reduced glutathione, a highly abundant thiol compound with potent reducing properties found in most cells. Its pivotal role involves regulating cellular control over reactive oxygen species. GST is an important detoxification enzyme in organisms where it can catalyze the combination of glutathione with various substrates. In addition, it plays a key role in the antioxidant system.

**Figure 3:**
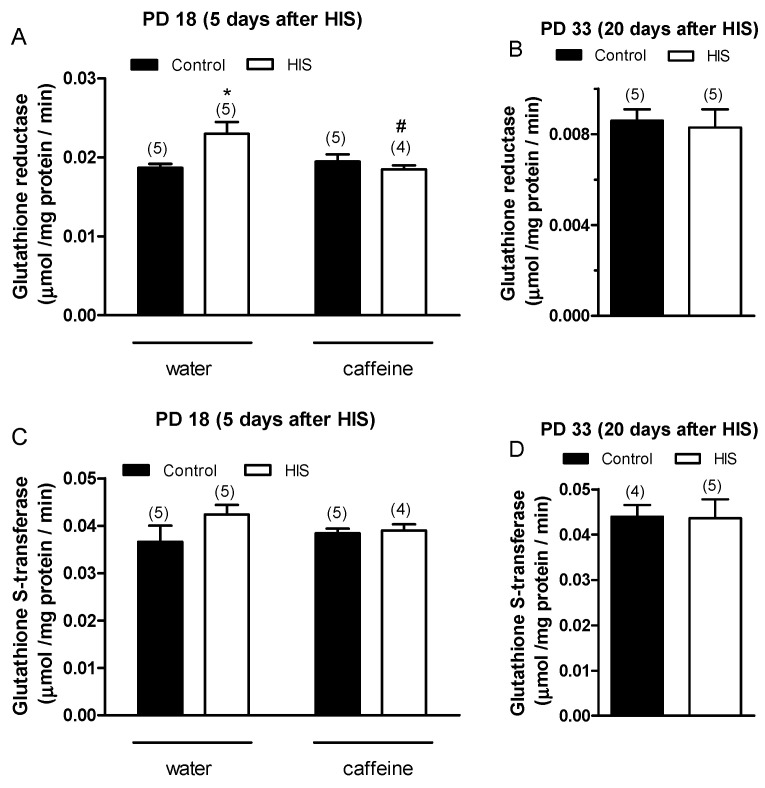
The impact of hyperthermia-induced seizures (HIS) and maternal caffeine consumption (1 g/L) during gestation and lactation on the activities of glutathione reductase (GR) and glutathione-S-transferase (GST) in the neonatal cortex (PD18 and PD33) was investigated at 5 and 20 days post HIS. The bar graphs illustrate the influence of HIS on GR (**A**,**B**) and GST (**C**,**D**) activities measured in the cortex of neonates born to mothers who consumed either water or caffeine during gestation and lactation. The data represent mean ± S.E.M. values of 4–5 neonates (in parentheses) from independent litters. * *p* < 0.05 signifies a significant difference from the corresponding water control group, and # *p* < 0.05 denotes a significant difference from the water HIS group, determined through a two-way ANOVA and Bonferroni post hoc test. The control group comprises pups not exposed to hyperthermia-induced seizures but separated from the dams for the same duration and placed in a chamber at room temperature. HIS refers to pups subjected to hyperthermia-induced seizures as described in Methods.

Two-way ANOVA analysis revealed that there was a significant effect in the interaction between HIS and caffeine [F(1,15) = 7.15, *p* = 0.017] on GR activity 5 days after hyperthermic seizures. However, we did not find any significant effect on GR activity in the HIS [F(1,15) = 2.8, *p* = 0.11] caffeine [F(1,15) = 3.5, *p* = 0.081] groups. The Bonferroni post-test showed that HIS significantly increased GR activity in control rats (*p* < 0.05), and this effect was significantly blocked by caffeine (*p* < 0.05) (Figure 3A). On the other hand, the effect of HIS on-GR activity disappeared 20 days after hyperthermic seizures, similar to what was observed in TBARS (Figure 3B).

Concerning GST activity, statistical analyses did not reveal any significant effect of HIS and maternal caffeine intake either at 5 days (Figure 3C) or at 20 days after hyperthermic seizures (Figure 3D). Our focus was on analyzing whether caffeine could reverse the changes in GR and GST activity induced by HIS. Since after 20 days, we did not observe any differences between the control samples and those exposed to HIS, we decided not to include the two groups treated with caffeine.

### 3.4. Effect of HIS and Maternal Caffeine Intake during Gestation and Lactation on Na^+^/K^+^-ATPase Activity in Cortex Brain

**Figure 4:**
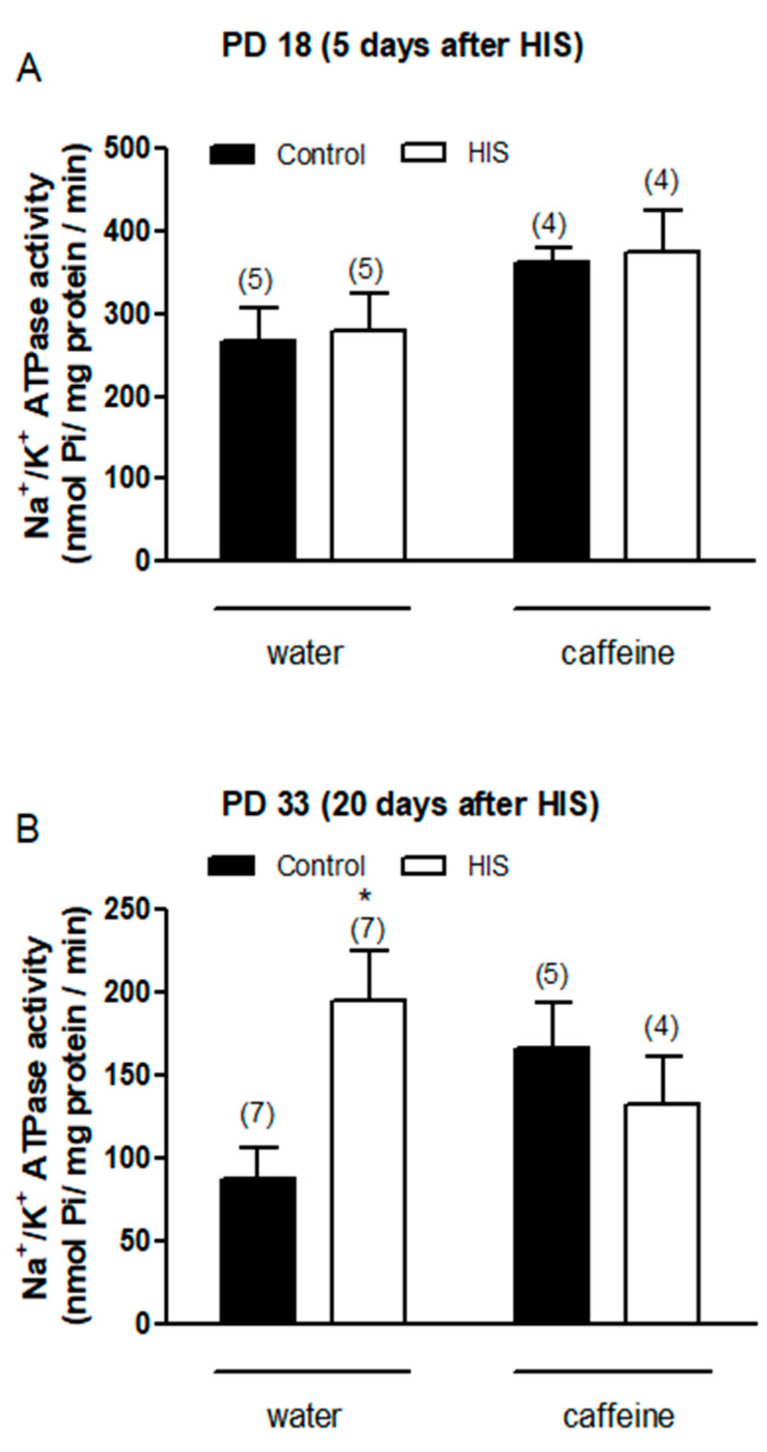
The impact of hyperthermia-induced seizures (HIS) and maternal caffeine consumption (1 g/L) during gestation and lactation on Na^+^/K^+^-ATPase activity was assessed in the neonatal cortex (PD18 and PD33) at 5 (**A**) and 20 days (**B**) post hyperthermia-induced seizures. Bar graphs illustrate the effect of HIS on Na^+^/K^+^-ATPase activity measured in the cortex of neonates born to mothers who consumed either water or caffeine during gestation and lactation. Data represent mean ± S.E.M. values from 4 to 7 neonates (in parentheses) in independent litters. * *p* < 0.05 indicates a significant difference from the corresponding water control group, as determined with two-way ANOVA and Bonferroni post hoc test. The control group consists of pups not exposed to hyperthermic stimulus but separated from the dams for the same duration and placed in a chamber at room temperature. HIS refers to pups exposed to hyperthermia-induced seizures as described in Methods.

As shown in Figure 4A, the statistical analyses carried out 5 days after hyperthermic insult found a significant effect of maternal caffeine intake [F(1,14) = 5.5, *p* = 0.03] but did not find any significant effect of HIS [F(1,14) = 0.09, *p* = 0.77] or HIS × caffeine interaction [F(1,14) = 0.0, *p* = 0.98] on the Na^+^/K^+^-ATPase activity. The Bonferroni post-test did not find any significant variation when the effect of caffeine was assayed in both control rats (control-water vs. control-caffeine, *p* > 0.05) and submitted to HIS (HIS-water vs. HIS-caffeine, *p* > 0.05).

Concerning the effect of HIS and maternal caffeine intake on the Na^+^/K^+^-ATPase activity 20 days after hyperthermic insult, two-way ANOVA analyses found a significant interaction between HIS and caffeine [F(1,19) = 6.95, *p* = 0.016], whereas no significant effect of HIS [F(1,19) = 1.88, *p* = 0.186] and maternal caffeine intake [F(1,19) = 0.09, *p* = 0.76] was detected. The Bonferroni post-test showed that HIS significantly increased the activity of Na^+^/K^+^ ATPase level in control rats (*p* < 0.05) (Figure 4B). Indeed, two-way ANOVA analyses revealed that there was a significant interaction between HIS and maternal caffeine consumption. However, when we applied Bonferroni´s post-test to compare the groups HIS vs. HIS caffeine, no significant difference was found (Figure 4B).

### 3.5. Effect of HIS and Maternal Caffeine Intake during Gestation and Lactation on Mg^2+^-ATPase Activity in Cortex Brain

**Figure 5:**
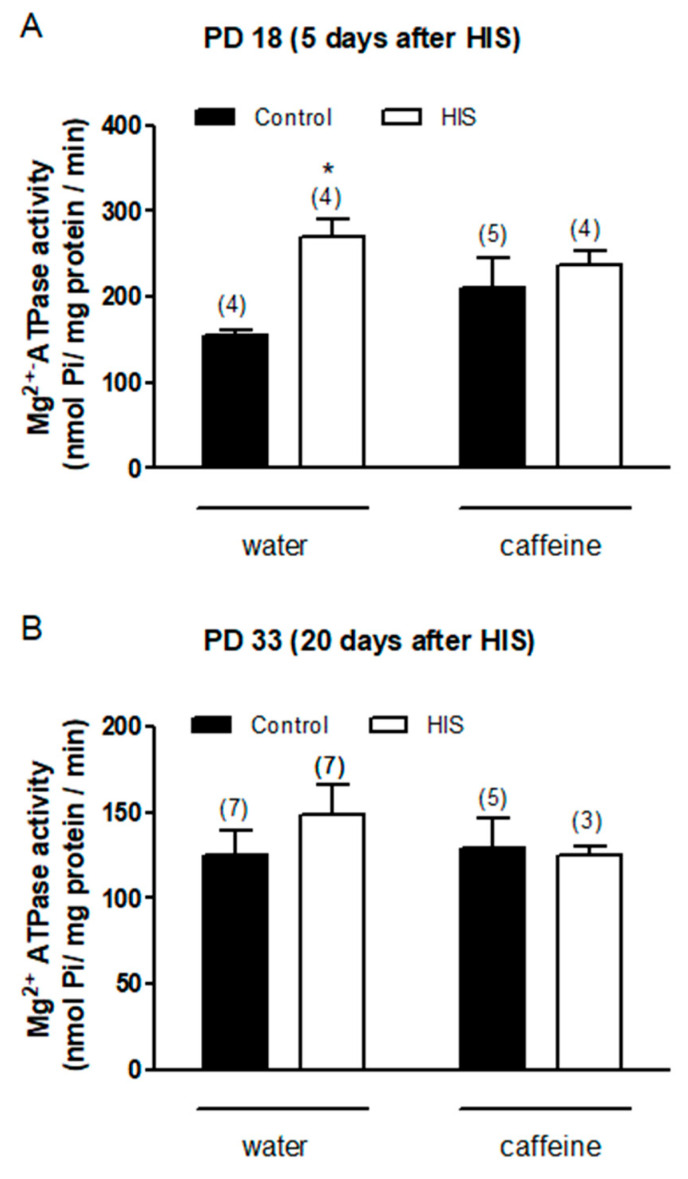
The impact of hyperthermia-induced seizures (HIS) and maternal caffeine consumption (1 g/L) during gestation and lactation on Mg^2+^-ATPase activity was investigated in the neonatal cortex (PD18 and PD33) at 5 (**A**) and 20 days (**B**) post hyperthermia-induced seizures. Bar graphs depict the effect of HIS on Mg^2+^-ATPase activity measured in the cortex of neonates born to mothers who consumed either water or caffeine during gestation and lactation. Data represent mean ± S.E.M. values from 3 to 7 neonates (in parentheses) in independent litters. * *p* < 0.05 indicates a significant difference from the corresponding water control group, determined through a two-way ANOVA and Bonferroni as a post hoc test. The control group comprises pups not subjected to hyperthermic stimulus but separated from the dams for the same duration and placed in a chamber at room temperature. HIS refers to pups exposed to hyperthermia-induced seizures using a warmed air stream (45–50°) from a hair dryer.

We also wanted to learn about the effect of HIS and chronic maternal caffeine intake on Mg^2+^-ATPase activity. The two-way ANOVA analyses revealed that 5 days after hyperthermic insult, HIS caused a significant change in Mg^2+^-ATPase activity in the control group [F(1,13) = 7.75, *p* = 0.0155]. However, statistical analyses did not find any significant effect of maternal caffeine intake [F(1,13) = 0.19, *p* = 0.67] or caffeine × HIS interaction [F(1,13) = 3.06, *p* = 0.104]. The Bonferroni post-test showed that HIS significantly increased the activity of Mg^2+^-ATPase level in control rats (*p* < 0.05) (Figure 5A).

No significant effects were observed when Mg^2+^-ATPase activity was studied 20 days after HIS. Thus, the ANOVA two-way analyses failed to find any significant effect of HIS [F(1,18) = 0.31, *p* = 0.58], caffeine [F(1,18) = 0.31, *p* = 0.58], and caffeine × HIS interaction [F(1,18) = 0.61, *p* = 0.44] (Figure 5B).

## 4. Discussion

### 4.1. HIS Induce Oxidative Stress in the Medium Term in Cortex Brain

**Figure 6:**
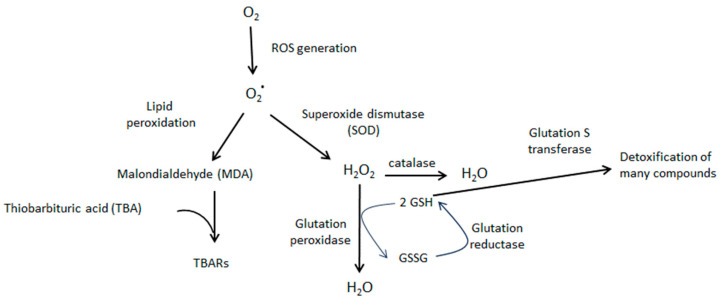
Schematic diagram showing the cascade of oxidative stress and the role played by TBARs, glutathione reductase, and glutathione S transferase. O_2_^*^ represents superoxide ion.

The obtained results suggest that hyperthermic insults induce oxidative stress in the cortex brain, which is detectable even after 5 days of hyperthermic insult. Thus, the TBARS level, which is considered a good parameter of oxidative stress within a biological sample, significantly increased 5 days after HIS (PD 18) [18]. However, this effect was transitory since it disappeared 20 days after HIS (PD 33). Previous work carried out in our laboratory showed a significant loss of GR and GST activities in cortex rats after 48 h of HIS [19]. These enzymes are involved in protection against oxidative stress [20]. Therefore, the increase in TBARS level observed 5 days after HIS could be explained by the significant loss of GR and GST activities detected 48 h after HIS. In accordance with this hypothesis, the observed increase in GR activity 5 days after HIS could be attributed to a compensatory mechanism aimed at counteracting the production of free radicals. Therefore, we hypothesize that there is a time lag between alterations in glutathione reductase (GR) and glutathione S-transferase (GST) activity and the subsequent emergence of elevated TBAR levels. We propose that initially (48 h after heat-induced stress—HIS), a reduction in GR and GST activity occurs. These diminished levels of antioxidant enzyme activity lead to oxidative stress, manifested by an increase in TBAR levels. We posit that, in response to the heightened oxidative stress, the cell initiates a compensatory mechanism by elevating GR activity. However, this increase does not immediately impact TBAR levels; rather, it takes several hours or days for this parameter to return to normal. In our observations, we have noted a normalization of TBAR levels 20 days after HIS, but it is plausible that normalization could occur earlier. Although a growing body of evidence suggests that oxidative stress may play a key role in the epileptogenic process [21], there is limited information about the presence of oxidative stress in brain areas directly involved in epileptogenesis in febrile seizures. Lipid peroxidation is a common consequence of oxidative stress. Thus, lipid peroxidation is a biochemical process in which free radicals, such as reactive oxygen species (ROS), target the double bonds present in lipids. As a result of this process, various compounds, including malondialdehyde (MDA), are generated. MDA readily reacts with thiobarbituric acid (TBA), producing MDA-TBA2 conjugates known as TBARs, which exhibit absorbance at 535 nm. Conversely, ROS can be converted into hydrogen peroxide through the action of superoxide dismutase. Hydrogen peroxide can then be detoxified by both catalase and glutathione peroxidase activities. The latter requires reduced glutathione, which is generated through the action of glutathione reductase. Finally, reduced glutathione is also utilized by glutathione S-transferase to detoxify various compounds (Figure 6).

Recently, Zaniani and coworkers (2022) showed that the malondialdehyde (MDA) level increased significantly 6 h after HIS [22]. Similar results have been shown in humans, where biochemical analyses of blood samples demonstrated a significant increase in the oxidant MDA 2–8 h after febrile seizures [23,24]. However, these studies did not analyze the persistence of oxidative stress following hyperthermic insult. Our results have shown that oxidative stress was still present 5 days after HIS. This may be especially interesting since the consequences of oxidative stress depend on the intensity of its production [21].

### 4.2. Maternal Caffeine Intake Promotes Beneficial Effects in Neonates Submitted to HIS

Our results suggest that chronic oral caffeine intake during gestation and lactation abolished hyperthermic seizure-induced oxidative stress. Thus, caffeine intake eliminated both the increase observed in TBARS level 5 days after HIS and the variations in the activity of GR. A previous study carried out in our laboratory showed that caffeine was also able to abolish oxidative stress induced by HIS in the cerebellum [15]. Therefore, with the present study, we expand our understanding of the positive effects resulting from maternal caffeine consumption, providing evidence that caffeine can also reverse oxidative stress in the cerebral cortex following neonatal hyperthermia seizures. However, it is also important to consider that maternal caffeine intake alters two parameters of neonatal development, pinna opening and righting reflex on the surface.

Concerning the effect of HIS and caffeine intake on Na^+^/K^+^-ATPase activity, statistical analysis revealed a significant interaction between the two factors. Therefore, it is tempting to speculate that oxidative stress is the underlying cause of the variation in Na^+^/K^+^-ATPase activity. Supporting this hypothesis, previous studies have shown that free radicals generated during oxidative stress may act as signaling messengers, which regulate Na^+^/K^+^-ATPase activity in the Central Nervous System [25,26]. Thus, the mechanism proposed suggests that the subunits of the pump present cysteine residues, which are the targets of free radicals. As a result of the interaction between cysteine residues and free radicals, mixed disulfates are formed, which can evoke a loss of Na^+^/K^+^-ATPase activity [26]. Taking into account that Na^+^/K^+^-ATPase plays a key role in the control of neuronal excitability, the increase in Na^+^/K^+^-ATPase activity observed in this work could be explained as a compensatory mechanism.

In the case of Mg^2+^-ATPase, the results obtained have shown that there is no significant interaction between HIS and caffeine. Therefore, we think that the increase observed in the activity of this pump 5 days after hyperthermic insult was not related to the oxidative stress induced by HIS.

**Figure 7:**
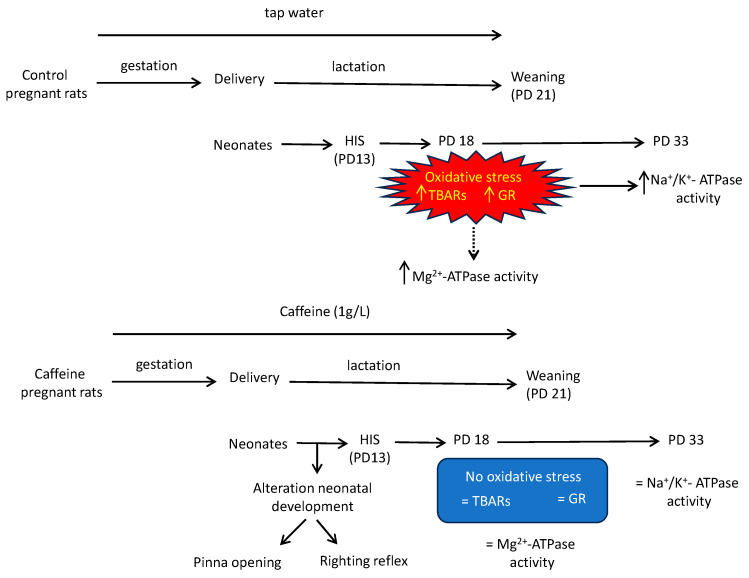
Schematic representation of the main findings in this study. Two groups of pregnant rats were used in this study: the first group received caffeine (1 g/L) in their drinking water from gestational day 2 throughout gestation and lactation whereas the second drank tap water. On postnatal day 13, half of each litter was subjected to hyperthermia-induced seizures (HIS), while the other half served as controls. Five (PD18) and twenty days after the hyperthermic insult (PD33), the rats were euthanized, and their cortical brains were removed to investigate the effects of maternal caffeine consumption and hyperthermia-induced seizures on oxidative stress parameters, Na^+^/K^+^-ATPase, and Mg^2+^-ATPase activities. In control groups, HIS evoked oxidative stress at PD 18 and, as a consequence, a loss of Na^+^/K^+^-ATPase activity. These effects were avoided when maternal rats drank caffeine during gestation and lactation. However, caffeine altered two parameters of neonatal development, which must also be considered. ↑ means increase activity; ↓ means decrease activity; = means that activity remains unchanged; dot line means that we think that the increase observed in the activity of this pump 5 days after hyperthermic insult was not related to the oxidative stress induced by HIS.

Taken together, the elimination of oxidative stress and the restoration of Na^+^/K^+^-ATPase activity suggest that maternal caffeine intake during gestation and lactation may have beneficial effects on neonates with febrile seizures. Supporting this hypothesis, our previous study revealed that the average temperature at which stage 5 seizures began was significantly higher in neonates exposed to maternal caffeine compared to the control group (42.1 ± 0.4 vs. 40.4 ± 0.8, *p* < 0.05). However, we cannot disregard the effects of maternal caffeine intake on the pinna opening and surface righting reflex. Thus, righting and geotaxis reflexes are utilized as indicators of early developmental stages in newborns [27]. The findings from this study demonstrate that although the geotaxis reflex remained unaffected, neonates exposed to maternal caffeine exhibited a significant delay in the righting reflex. Something similar happens with the pinna and eye-opening parameters, which are used as indicators of morphological development. Our results have shown a slight but significant delay in pinna opening, although the eye opening remained unaltered (Figure 7).

Therefore, as hyperthermia at early ages is related to epilepsy in adults, the antioxidant effect of caffeine exposure during early age could avoid the potential damage in adults, although additional study is needed to evaluate the impact of the developmental alteration parameters in newborns on their adult brain development.

## Data Availability

Data is contained within the article.

## References

[1] Frary C.D., Johnson R.K., Wang M.Q. (2005). Food Sources and Intakes of Caffeine in the Diets of Persons in the United States. J. Am. Diet. Assoc..

[2] Brazier J.L., Ritter J., Berland M., Khenfer D., Faucon G. (1983). Pharmacokinetics of Caffeine during and after Pregnancy. Dev. Pharmacol. Ther..

[3] Fredholm B.B., Bättig K., Holmén J., Nehlig A., Zvartau E.E. (1999). Actions of Caffeine in the Brain with Special Reference to Factors That Contribute to Its Widespread Use. Pharmacol. Rev..

[4] Iglesias I., León D., Ruiz M.A., Albasanz J.L., Martín M. (2006). Chronic Intake of Caffeine during Gestation down Regulates Metabotropic Glutamate Receptors in Maternal and Fetal Rat Heart. Amino Acids.

[5] Lorenzo A.M., León D., Castillo C.A., Ruiz M.A., Albasanz J.L., Martín M. (2010). Maternal Caffeine Intake during Gestation and Lactation Down-Regulates Adenosine A1 Receptor in Rat Brain from Mothers and Neonates. J. Neurosci. Res..

[6] Ősz B.-E., Jîtcă G., Ștefănescu R.-E., Pușcaș A., Tero-Vescan A., Vari C.-E. (2022). Caffeine and Its Antioxidant Properties-It Is All about Dose and Source. Int. J. Mol. Sci..

[7] Tiwari V., Mishra A., Singh S., Shukla S. (2023). Caffeine Improves Memory and Cognition via Modulating Neural Progenitor Cell Survival and Decreasing Oxidative Stress in Alzheimer’s Rat Model. Curr. Alzheimer Res..

[8] Souza M.A., Mota B.C., Gerbatin R.R., Rodrigues F.S., Castro M., Fighera M.R., Royes L.F.F. (2013). Antioxidant Activity Elicited by Low Dose of Caffeine Attenuates Pentylenetetrazol-Induced Seizures and Oxidative Damage in Rats. Neurochem. Int..

[9] Endesfelder S., Weichelt U., Strauß E., Schlör A., Sifringer M., Scheuer T., Bührer C., Schmitz T. (2017). Neuroprotection by Caffeine in Hyperoxia-Induced Neonatal Brain Injury. Int. J. Mol. Sci..

[10] Shinnar S., Glauser T.A. (2002). Febrile Seizures. J. Child Neurol..

[11] Stafstrom C.E. (2002). Assessing the Behavioral and Cognitive Effects of Seizures on the Developing Brain. Prog. Brain Res..

[12] McClelland S., Dubé C.M., Yang J., Baram T.Z. (2011). Epileptogenesis after Prolonged Febrile Seizures: Mechanisms, Biomarkers and Therapeutic Opportunities. Neurosci. Lett..

[13] Dubé C.M., Brewster A.L., Baram T.Z. (2009). Febrile Seizures: Mechanisms and Relationship to Epilepsy. Brain Dev..

[14] Baram T.Z., Gerth A., Schultz L. (1997). Febrile Seizures: An Appropriate-Aged Model Suitable for Long-Term Studies. Brain Res. Dev. Brain Res..

[15] Crespo M., León-Navarro D.A., Martín M. (2018). Cerebellar Oxidative Stress and Fine Motor Impairment in Adolescent Rats Exposed to Hyperthermia-Induced Seizures Is Prevented by Maternal Caffeine Intake during Gestation and Lactation. Eur. J. Pharmacol..

[16] Crespo M., León-Navarro D.A., Martín M. (2022). Na^+^/K^+^- and Mg^2+^-ATPases and Their Interaction with AMPA, NMDA and D2 Dopamine Receptors in an Animal Model of Febrile Seizures. Int. J. Mol. Sci..

[17] Novaes L.S., Dos Santos N.B., Dragunas G., Perfetto J.G., Leza J.C., Scavone C., Munhoz C.D. (2018). Repeated Restraint Stress Decreases Na,K-ATPase Activity via Oxidative and Nitrosative Damage in the Frontal Cortex of Rats. Neuroscience.

[18] Aguilar Diaz De Leon J., Borges C.R. (2020). Evaluation of Oxidative Stress in Biological Samples Using the Thiobarbituric Acid Reactive Substances Assay. J. Vis. Exp..

[19] León Navarro D.A., Crespo M., Martín M. (2020). Oxidative Stress in Epileptogenesis: Febrile Seizures, Chemoconvulsant Pilocarpine, and Electrical Stimulation. Oxidative Stress and Dietary Antioxidants in Neurological Diseases.

[20] Rodríguez-Rodríguez A., Egea-Guerrero J.J., Murillo-Cabezas F., Carrillo-Vico A. (2014). Oxidative Stress in Traumatic Brain Injury. Curr. Med. Chem..

[21] Łukawski K., Czuczwar S.J. (2023). Oxidative Stress and Neurodegeneration in Animal Models of Seizures and Epilepsy. Antioxidants.

[22] Zaniani N.R., Roohbakhsh A., Moghimi A., Mehri S. (2022). Protective Effect of Toll-like Receptor 4 Antagonist on Inflammation, EEG, and Memory Changes Following Febrile Seizure in Wistar Rats. Behav. Brain Res..

[23] Güneş S., Dirik E., Yiş U., Seçkin E., Kuralay F., Köse S., Unalp A. (2009). Oxidant Status in Children after Febrile Seizures. Pediatr. Neurol..

[24] El-Masry H.M.A., Sadek A.A., Hassan M.H., Ameen H.H., Ahmed H.A. (2018). Metabolic Profile of Oxidative Stress and Trace Elements in Febrile Seizures among Children. Metab. Brain Dis..

[25] Silva L.F.A., Hoffmann M.S., Rambo L.M., Ribeiro L.R., Lima F.D., Furian A.F., Oliveira M.S., Fighera M.R., Royes L.F.F. (2011). The Involvement of Na^+^, K^+^-ATPase Activity and Free Radical Generation in the Susceptibility to Pentylenetetrazol-Induced Seizures after Experimental Traumatic Brain Injury. J. Neurol. Sci..

[26] Bogdanova A., Petrushanko I.Y., Hernansanz-Agustín P., Martínez-Ruiz A. (2016). “Oxygen Sensing” by Na,K-ATPase: These Miraculous Thiols. Front. Physiol..

[27] Gibb R., Kolb B. (2005). Neonatal Handling Alters Brain Organization but Does Not Influence Recovery from Perinatal Cortical Injury. Behav. Neurosci..

